# Determinants of severe acute malnutrition among children aged 6–59 months in the pastoral community of Liban District, Guji Zone, Oromia Regional State, Southeastern Ethiopia: a case–control study

**DOI:** 10.1017/jns.2021.98

**Published:** 2021-12-07

**Authors:** Diriba S. Gemechu, Yoseph Worku, Abebe Alemu, Urge Gerema

**Affiliations:** 1 Ethiopian Public Health Institute (EPHI), Center of Public Health Emergency Management (cPHEM), Addis Ababa, Ethiopia; 2 St. Paul's Hospital Millennium Medical College, Addis Ababa, Ethiopia; 3 International Medical Crops, Addis Ababa, Ethiopia; 4 Department of Biomedical Sciences, College of Medical Sciences, Institute of Health Sciences, Jimma University, Jimma, Ethiopia

**Keywords:** Determinants, Ethiopia, Severe acute malnutrition, Under five children, AOR, adjusted odds ratio, CI, confidence interval

## Abstract

Malnutrition remains one of the most common causes of morbidity and mortality among children, particularly in Ethiopia. The present study aimed to assess determinants of severe acute malnutrition among children aged 6–59 months in the pastoral community of Liban District, Southeastern Ethiopia. A case–control study design was conducted on 89 cases and 177 controls from 1–30 December 2020. A simple random sampling technique was used to select study participants. Data collected using interviewer-administered structured questionnaire were used, and anthropometric measurements were done by standardised calibrated instruments to collect data. Data were entered into EPI data version 3.1 and then exported to SPSS version 25.0 software for analysis. All candidate variables with *P* < 0⋅25 in bivariate analysis were then entered into multivariable logistic regression. Associated factors were identified at *P* < 0⋅05 and 95 % CI. A total of 266 (89 cases and 177 controls) having a response rate of 96⋅6 % being underweight [adjusted odds ratio (AOR) = 11⋅8, 95 % CI 3⋅17, 43⋅89], illness previous 2 weeks (AOR = 3⋅47, 95 % confidence interval (CI) 1⋅34, 8⋅99), family member with malnutrition (AOR = 4⋅52, 95 % CI 1⋅45, 14⋅01), greater than five family size, (AOR = 5⋅33, 95 % CI 2⋅08, 13⋅66), mothers unable to read and write (AOR = 3⋅66, 95 % CI 1⋅27, 10⋅56), mothers with low decision autonomy (AOR = 5⋅67, 95 % CI 2⋅26, 14⋅27), not handwashing at all critical time (AOR = 7⋅23, 95 % CI 2⋅74, 19⋅07), not feeding child animal source (AOR = 7⋅13, 95 % CI 1⋅98, 25⋅59), bottle feeding (AOR = 7⋅06, 95 % CI 2⋅34, 21⋅28) and being married (AOR = 0⋅05, 95 % CI 0⋅02, 0⋅19) were significantly associated with acute malnutrition. The present study has confirmed the association of acute malnutrition with maternal education, underweight, family size and inappropriate infant caring practices.

## Background

The term malnutrition is to both undernutrition as well as overnutrition. However, in maximum cases, the terms malnutrition and protein–energy malnutrition (PEM) are used interchangeably with undernutrition^(1)^. PEM applies to a group of related disorders that include marasmus, kwashiorkor and intermediate states of marasmic kwashiorkor. Three anthropometric indicators are commonly used to describe the nutritional status of the children: weight-for-height (wasting), height-for-age (stunting) and weight-for-age (underweight)^(2)^. A deficit in any of these reflects malnutrition and a *z*-score below −3 indicates a severe form of illness. Malnutrition limits the potential of a country and is strongly associated with mortality, morbidity, reduced cognitive performance and compromised productivity among its population^(1–4)^.

Healthy child growth and development are the basis of human development. The impact of malnutrition is multifarious. It has an all-pervasive impact on the physical well-being and socio-economic condition of a nation. It perpetuates poverty through direct losses in productivity; indirect losses from poor cognitive function, poor child development and deficits in schooling; and losses due to increased health costs^(2,5)^. PEM is more of a social problem like the biological translation of a social disease. PEM's basic causes stem from inadequate resources, poor resource utilisation and lack of control of resource. The compounding effects of both underlying and immediate causes are eventually responsible for its manifestation^(5,6)^.

Malnutrition remains one of the most common causes of morbidity and mortality among children under 5 years throughout the World^(2)^. Across the globe, an estimated 16 million children under the age of 5 years are affected by acute malnutrition^(7)^. This number is staggering most importantly because children with acute malnutrition are nine times more likely to die than well-nourished children^(2)^. These deaths are the direct result of malnutrition itself, as well as the indirect result of childhood illnesses like diarrhoea and pneumonia that malnourished children are too weak to survive. Over two-thirds of these deaths, which are often associated with inappropriate feeding practices, occur during the first year of life^(8–10)^. It has been responsible, directly or indirectly, for 60 % of the 10⋅9 million deaths annually among children^(11)^. Malnutrition is one of the leading causes of morbidity and mortality in children under 5 years of age in Ethiopia. The country has the second-highest rate of malnutrition in sub-Saharan Africa^(2,12,13)^.

Ethiopia would have only reached the Millennium Development Goal target of halving the number of underweight children if the percentage reduction is increased to at least 1⋅6 percentage points per year but failed^(14,15)^. Under the umbrella of the growth and transformation plan II, the government of Ethiopia launched the Health Sector transformation plan which gives due considerations for nutrition than the previous plans for reducing the prevalence of wasting from 11 to 3 %^(10)^. From 2004/5, the Federal Ministry of Health (FMoH), alongside partners including the United Nations Children's Fund (UNICEF) and others, commenced scale-up of severe acute malnutrition (SAM) treatment services^(16)^.

In 2007, following international endorsement of the CMAM approach, the national protocol for SAM treatment was revised to include detailed guidance for the Outpatient Therapeutic Programme (OTP) and community mobilisation activities^(17)^. However, the problem is still high in its magnitude and caused challenges for the attainment of the goals to reduce child mortality, especially in the Pastoral community. A huge number of sever acute malnutrition are reported from pastoral community areas; the predictors of the problem could not identify specifically the Liban District, Guji Zone. Some studies tried to quantify the determinants of SAM among the agrarian community, and very few studies were done in the pastoral community. Those studies also lack detailed community and household-level variables. On top of that Liban District is a pre-dominantly pastoral area known for its recurrent drought for several years, therefore, aimed to assess determinants of acute malnutrition among children aged 6–59 months in the pastoral community of Liban District, Guji Zone, Oromia Regional State, Southeastern Ethiopia. This is important to guide public health planners and policymakers to design appropriate strategies and interventions to enhance nutritional status in the pastoral community.

## Methods

### Study area and period

The study was conducted in the pastoral community of Liban District, Guji Zone, Oromia Regional State, Southeastern Ethiopia. It is located at a distance of 526 from Addis Ababa, capital city. Liban district is one of the pastoral districts found in the Guji Zone which has a total population of 79 322, of which 51 % are female and children under five years of age are 13 033. The study was conducted from 1 to 30 December 2019.

### Study design

An unmatched case–control study design was used to assess determinants of acute malnutrition among children aged 6–59 months in the pastoral community of Liban District, Guji Zone, Oromia Regional State, Southeastern Ethiopia.

### Population

Source populations were all children aged 6–59 months in the Liban District, Guji Zone who have been lived in the District for at least 6 months prior to data collection. The study populations were children aged 6–59 months who have been lived in the District and randomly included in the study either as cases or controls that fulfilled the inclusion criteria and resided in the district for at least 6 months.

### Eligibility criteria

#### Cases

Children aged 6–59 months who were diagnosed as SAM by fulfilling World Health Organization (WHO) criteria of SAM.

#### Controls

Controls were children without malnutrition diagnosed as WHO criteria of SAM. Both cases and controls are selected from the same residence. Both cases and controls were selected from the same kebeles.

### Exclusion criteria

Patients with known secondary malnutrition due to other causes like diabetes mellitus, hyperthyroidism, congenital anomalies, cerebral palsy and kidney disease will be excluded. Cases with known chronic illnesses like TB, HIV, cirrhosis of liver, protein-losing enteropathy and oedema due to congestive heart failure and congenital abnormality that can affect the feeding pattern of the child like congenital heart disease will be excluded.

### Operational definitions

#### Severe acute malnutrition

*Case*: A child aged 6–59 months is classified as malnourished, if she/he has one or more of the following: (1) weight-for-height *z*-score (WHZ) <−3 sd; (2) middle-upper arm circumstance (MUAC) <115 mm; (3) bilateral nutritional pitting.

*Control*: A child with MUAC ≥12⋅5, WHZ >−2 sd and without bilateral pitting oedema of nutritional origin.

*Diarrhoea*: A child having three or more loose or watery stools per day.

*Acute respiratory infection*: A child with cough, fast breathing or difficulty in breathing and fever.

### Sample size determination and sampling procedure

Sample size calculated using double population proportion formulae by using EPI Info version 7. The assumptions and the parameters used for sample size calculation are detecting: proportion of suboptimal infant feeding practices among the cases 0⋅34 and 0⋅68 among controls^(18)^; proportion of having >5 family size among the cases 0. 49 and 0⋅68 among controls^(18)^; proportion of having diarrhoea among the cases 0⋅07 and 0⋅27 among the controls^(19)^ and proportion of complementary feeding <2 times per day among the cases 0⋅26 and 0⋅43 among controls^(12)^. A 95 % confidence interval (CI), 80 % power and the ratio of control to case of 2:1 were applied. The different sample size was found using different related variables, and the largest sample size was taken. Therefore, the obtained sample size was 81 cases and 161 controls after adding 10 % expected non-response rate on each, and the total final sample size was 266. Six kebeles (the smallest administrative unit in Ethiopia next to district) were randomly selected from a total of 12 kebeles of Liban District, Guji Zone. Then the sample size was allocated proportionally to each kebele based on the household of each kebele. Study participants from the kebeles were randomly selected. After children with malnutrition (cases) aged 6–59 months were selected and mother interviewed, two normal children (controls) aged 6–59 months were selected from the same kebeles until the required sample size was obtained.

## Data collection tools

A structured and interviewer-administered questionnaire was used to obtain information from the children's mother/caregiver. For each child either case or control, a detailed history nutritional status, birth history, immunisation status, socio-demographic, maternal education and family income of parents were recorded, and anthropometric measurements were done subsequently. To maintain data quality, the questionnaire was developed in English and translated into the local language (Afan Oromo). Data collectors who can speak local language and health professional were recruited and trained for 1 d. All equipment used to measure the variables were readily available to collect data. The interview took 20–25 min.

### Anthropometric measurement

Anthropometric measurement of the children was measured based on the WHO standardised procedures. Weight and recumbent length/ height were taken according to WHO standardised techniques^(20)^. Validation of instruments, and measurements and random auditing were done on a daily basis. Trained health professionals were taken the anthropometric measurements in the houses of the selected children during the daytime.

### Data quality assurance

To ensure the quality of data, the questionnaire was pre-tested on 5 % of the actual sample size in another Woreda (Guji Zone) before the actual data collection. After analysing pre-test results, necessary modifications and corrections were made. Every day the collected data were checked for completeness. Consequently, amendments and corrections were made.

### Data processing and data analysis

The collected data were checked for its completeness and coded, entered into EPI data version 3.1, and then exported to SPSS version 25 for analysis. The anthropometric data were analysed. Descriptive statistics such as frequencies, percentages and means were computed as necessary. The bivariate and multivariable logistic regression model was used to determine the degree of association between the outcome and predictor variables. Variables having a *P*-value of <0⋅25 in the bivariate model were subjected to multivariable analysis to avoid confounding variables’ effect. Multicollinearity was checked, and the goodness of fit of the multivariate model was checked with the Hosmer and Lemeshow test (*P* = 0⋅32). Lastly, adjusted odds ratio (AOR) with 95 % CI and *P*-value < 0⋅05 was taken as statistically significant

## Results

### Socio-demographic characteristics of study participants

In a total of 266 children (89 cases and 177 controls) having a response rate of 96⋅6%, half of study participants, 46 (50⋅3 %) of cases and 96 (54⋅2 %) of controls, were female. About 75 % of both cases and controls were below 36 months of age, and 50 % were below 24 months of age. About 51 % of case's households and 36 % of control's households have greater than five family sizes. The mean weight of cases was 6⋅7 (sd ± 2⋅74) and 8⋅9 (sd ± 3⋅04) for controls. Regarding maternal educational status, about 67(75⋅3 %) of cases and 122(63⋅5 %) of controls were can not read and write, respectively. About two-thirds of case's households 67 (75⋅3 %) and 114 (64 %) of control's households earn <50 USD monthly income. The majority of the mothers, 77 (86⋅5 %) of the cases and 165 (93⋅2 %) of the controls, were housewives with occupation. Regarding the sex of the child, about 43 (48⋅3 %) of cases were male and 81 (45⋅8 %) of controls were male (Table 1).

**Table 1.** Socio-demographic characteristics of respondents in the pastoral community of Liban District, Guji Zone, Oromia Regional State, Southeastern Ethiopia, 2020

Variable		Acute malnutrition		COR (95 % CI)	*P*-value
		Control, *N* (%)	Case, *N* (%)		
Religion	Muslim	131 (74⋅0)	64 (71⋅9)	1⋅731 (0⋅642, 4⋅667)	0⋅278
	Protestant	16 (9⋅0)	12 (13⋅4)	1⋅180 (0⋅578, 2⋅411)	0⋅649
	Wakefata	30 (16⋅9)	13 (14⋅6)	1	
Maternal marital status	Married	103 (58⋅2)	71 (79⋅7)	0⋅339 (0⋅187, 0⋅614)	0⋅000
	Unmarried	74 (41⋅8)	18 (20⋅3)	1	
Maternal education	Illiterate	120 (67⋅8)	73 (82⋅1)	2⋅256 (1⋅21, 4⋅213)	0⋅011
	Literate	57 (32⋅2)	16 (17⋅9)	1	
Maternal occupation	Pastoral	12 (6⋅8)	12 (13⋅5)	485 (0⋅209, 1⋅127)	0⋅093
	Housewife	165 (93⋅2)	77 (86⋅5)	1	
Family size	5	36 (20⋅3)	43 (48⋅3)	3⋅917 (2⋅26, 6⋅779)	0⋅000
	≥5	141 (79)	46 (51⋅6)	1	
Maternal decision autonomy	Low level	74 (41⋅8)	57 (64⋅1)	2⋅610 (1⋅54, 4⋅402)	0⋅000
	Highest	103 (58⋅2)	32 (35⋅9)	1	
Sex of the child	Male	81 (45⋅8)	43 (48⋅3)	0⋅961 (0⋅580, 1⋅593)	0⋅879
	Female	96 (54⋅2)	46 (51⋅7)	1	
Paternal education	Illiterate	122 (63⋅5)	67 (75⋅3)	1⋅434 (0⋅807, 2⋅550)	0⋅219
	Literate	55 (71⋅4)	22 (24⋅7)	1	
Monthly income	<50USD	114 (64⋅4)	67 (75⋅3)	1⋅758 (0⋅995, 3⋅107)	
	≥50USD	63 (35⋅6)	22 (24⋅7)	1	0⋅052

COR, crude odds ratio.

### Child caring practices and maternal health services

More than half of case's mothers 54 (60⋅6 %) and 77 (43⋅5 %) of %) of control's mothers had exclusively breastfed their children for more than 6 months. Regarding the frequency of feeding, 38 (42⋅6 %) of case's mothers and 48 (27⋅1 %) of control's mothers fed their child <3 times/day which is suboptimal. After the age of 6 months, the majority of the cases 58 (65⋅1 %) and controls 119 (67⋅2 %) had initiated complementary feeding by cow milk. More than three-quarters of case children 71 (79⋅8 %) and 156 (88⋅1 %) control children received vitamin A supplementation at least once in their life (Table 2).

**Table 2.** Child caring practices and maternal health services in the pastoral community of Liban District, Guji Zone, Oromia Regional State, Southeastern Ethiopia, 2020

Variable		Acute malnutrition		COR (95 %CI)	*P*-value
		Control, *N* (%)	Case, *N* (%)		
Immunisation Status	Vaccinated for age	46 (26⋅0)	25 (28⋅1)	1⋅36 (0⋅742, 0⋅487)	0⋅324
	Not vaccinated	7 (4⋅0)	4 (4⋅4)	1⋅371 (0⋅38, 0⋅95)	0⋅629
	Incomplete	28 (15⋅8)	21 (23⋅5)	1⋅87 (0⋅97, 3⋅68)	0⋅063
	Fully vaccinated	96 (54⋅2)	39 (43⋅8)	1⋅00	
Exclusive breast feeding	≤6 months	100 (56⋅4)	35 (39⋅4)	2⋅13 (1⋅2, 3⋅67)	0⋅007
	>6 months	77 (43⋅5)	54 (60⋅6)	1⋅00	
Vitamin A supplementation	No	21 (11⋅9)	18 (20⋅2)	0⋅55 (0⋅28, 1⋅10)	0⋅092
	Yes	156 (88⋅1)	71 (79⋅8)	1⋅00	
Time of initiation of breast feeding	Late	55 (31⋅1)	30 (33⋅7)	1⋅07 (0⋅63, 1⋅84)	0⋅797
	Within 1 h	122 (68⋅9)	59 (67⋅4)	1⋅00	
Complementary food started	Adult food	43 (24⋅3)	22 (24⋅7)	0⋅97 (0⋅37, 2⋅54)	0⋅949
	Cow milk	119 (67⋅2)	58 (65⋅1)	0⋅81 (0⋅34, 1⋅96)	0⋅645
	Soup	15 (8⋅5)	9 (10⋅1)	1⋅00	
Feeding the child animal source food	No	47 (26⋅6)	40 (44⋅9)	2⋅47 (1⋅33, 4⋅60)	0⋅004
	Yes, less frequently	59 (33⋅3)	25 (28⋅0)	1⋅25 (0⋅65, 2⋅39)	0⋅498
	Yes, daily	71 (40⋅1)	24 (26⋅9)	1⋅00	
Feeding child vegetables and fruit	No	140 (79⋅1)	70 (78⋅6)	1⋅27 (0⋅38, 4⋅18)	0⋅697
	Yes, less frequently	26 (14⋅7)	15 (16⋅8)	1⋅54 (0⋅41, 5⋅74)	0⋅521
	Yes, once a week	10 (5⋅6)	3 (3⋅3)	1⋅00	
Frequency of feeding/ day	<3 times/day	48 (27⋅1)	38 (42⋅6)	1⋅89 (1⋅11, 3⋅22)	0⋅019
	≥3 times/day	129 (72⋅9)	51 (57⋅4)	1⋅00	
Material to feed child	Bottle	34 (19⋅2)	29 (32⋅5)	2⋅17 (1⋅18, 3⋅98)	0⋅012
	Cup	37 (20⋅9)	18 (20⋅2)	1⋅26 (0⋅66, 2⋅44)	0⋅482
	Hand	106 (59⋅9)	42 (47⋅2)	1⋅00	
Index child antenatal care	No	62 (35⋅0)	38 (42⋅6)	0⋅73 (0⋅44, 1⋅23)	0⋅237
	Yes	115 (65⋅0)	51 (57⋅3)		
Family planning	No	133 (75⋅1)	67 (75⋅2)	1⋅01 (0⋅56, 1⋅80)	0⋅980
	Yes	44 (24⋅9)	22 (24⋅7)	1⋅00	

COR, crude odds ratio.

Regarding the morbidity status of the children, 18 (20⋅2 %) of the cases and 17 (9⋅6 %) of the controls had diarrhoea 2 weeks before the study. Similarly, 15 (16⋅6 %) of the cases and 13 (7⋅3 %) of the controls had fever 2 weeks preceding the study. Accordingly, 52 (58⋅5 %) of cases and 124(70⋅1 %) of controls had the previous history of malnutrition with 51 (57⋅3 %) of cases and 147 (83⋅1 %) of controls had a family member with malnutrition. About 56 (62⋅9 %) of cases and 82 (46⋅3 %) of controls were stunted, whereas 72 (80⋅9 %) of cases and 78 (44⋅1 %) were underweight (Table 3).

**Table 3.** Child characteristics of study participants in the pastoral community of Liban, District, Guji Zone, Oromia Regional State, Southeastern Ethiopia, 2020

Variable		Acute malnutrition		COR (95 % CI)	*P*-value
		Case, *N* (%)	Control, *N* (%)		
Handwashing practice	Not at all critical time	46 (51⋅6)	52 (29⋅4)	2⋅62 (1⋅56, 4⋅42)	0⋅000
	at all critical time	43 (48⋅4)	125 (70⋅6)	1⋅00	
Staple food	Maize source food	36 (40⋅4)	63 (35⋅6)	1⋅01 (0⋅56, 1⋅83)	0⋅962
	Wheat source food	17 (19⋅1)	33 (18⋅6)	0⋅94 (0⋅460, 1⋅93)	0⋅871
	Milk product source	4 (4⋅4)	24 (13⋅6)	0⋅29 (0⋅09, 0⋅90)	0⋅033
	>Mixed of two or more source	32 (35⋅9)	57 (32⋅2)	1⋅00	
Water source	Unprotected source	47 (52⋅8)	82 (46⋅3)	1⋅32 (0⋅79, 2⋅18)	0⋅281
	Protected source	42 (47⋅1)	95 (53⋅7)	1⋅00	
Time to fetch water	≥30	65 (73⋅1)	72 (40⋅7)	0⋅54 (0⋅31, 0⋅94)	0⋅030
	<30 min	24 (26⋅9)	105 (59⋅3)	1⋅00	
House condition	Non ventilated	70 (78⋅6)	128 (72⋅3)	1⋅47 (0⋅81, 2⋅68)	0⋅210
	Ventilated	19 (21⋅3)	49 (27⋅7)	1⋅00	
Livestock ownership	No	5 (5⋅6)	14 (7⋅9)	1⋅23 (0⋅46, 3⋅32	0⋅681
	Yes	84 (94⋅4)	163 (92⋅1)	1⋅00	
Land ownership	No	10 (11⋅2)	23 (13⋅0)	1⋅10 (0⋅51, 2⋅37)	
	Yes	79 (88⋅8)	154 (87⋅0)	1⋅00	
Availability of latrine	No	16 (17⋅9)	28 (15⋅8)	0⋅83 (0⋅43, 1⋅61)	
	Yes	73 (82⋅1)	149 (84⋅2)	1⋅00	

### Environmental health-related factors

More than half of case's mothers 46 (51⋅6 %) and 52 (29⋅4 %) of control's mothers do not wash their hands at all critical times. The staple food of the area is Maize source food followed by wheat source foods. Most of the cases and controls, children households own livestock which account 84 (94⋅4 %) and 163 (92⋅1 %), respectively (Table 4).

**Table 4.** Environmental health conditions of study participants in the pastoral community of Liban District, Guji Zone, Oromia Regional State, Southeastern Ethiopia, 2020

Variable		Acute malnutrition		COR (95 % CI)	*P*-value
		Control, *N* (%)	Case, *N* (%)		
Previous history of malnutrition	Yes	124 (70⋅1)	52 (58⋅5)	1⋅72 (1⋅02, 2⋅91)	0⋅042
	No	53 (29⋅9)	37 (41⋅5)	1⋅00	
Family member with malnutrition	Yes	147 (83⋅1)	51 (57⋅3)	3⋅61 (2⋅04, 6⋅37)	0⋅000
	No	30 (16⋅9)	38 (42⋅7)	1⋅00	
Illness within 2 weeks	Yes	44 (24⋅9)	42 (47⋅2)	2⋅77 (1⋅62, 4⋅72)	0⋅000
	No	133 (75⋅1)	47 (52⋅8)	1⋅00	
Diarrhoea	Yes	17 (9⋅6)	18 (20⋅2)	2⋅28 (1⋅12, 4⋅69)	0⋅024
	No	160 (90⋅4)	71 (79⋅7)	1⋅00	
Fever	Yes	13 (7⋅3)	15 (16⋅6)	2⋅45 (1⋅12, 5⋅42)	0⋅026
	No	164 (92⋅7)	74 (83⋅4)	1⋅00	
Cough	Yes	14 (7⋅9)	10 (11⋅2)	1⋅58 (0⋅68, 3⋅64)	0⋅281
	No	163 (92⋅1)	79 (88⋅7)	1⋅00	
Weight for age (WFA) category	Underweight	78 (44⋅1)	72 (80⋅9)	4⋅55 (2⋅45, 8⋅45)	0⋅000
	Moderate underweight	31 (17⋅5)	10 (11⋅2)	2⋅24 (1⋅03, 4⋅84)	0⋅041
	Normal	68 (38⋅4)	7 (7⋅8)	1⋅00	
Height for age	stunting	82 (46⋅3)	56 (62⋅9)	2⋅56 (1⋅42, 4⋅61)	0⋅002
	Moderate stunting	19 (10⋅7)	12 (13⋅4)	2⋅28 (0⋅96, 5⋅45)	0⋅062
	Normal	76 (43⋅0)	21 (23⋅6)	1⋅00	

### Determinants of acute malnutrition

In bivariate analysis: variables like maternal marital status, family size, maternal decision autonomy, family member with malnutrition, illness in the last 2 weeks, being underweight and handwashing practice of mothers, maternal education, duration of exclusive breastfeeding, frequency of feeding per day, being stunted, not feeding the child animal source, material used to feed child, illness with diarrhoea and fever, time to fetch water at (*P* < 0⋅25, 95 % CI).

Finally, the variables that showed association at *P*-value < 0⋅05 on multivariable logistic regression were taken as the determinants of SAM. Accordingly, the results of multivariable logistic regression analyses being underweight (AOR = 11⋅8, 95 % CI 3⋅17, 43⋅89), illness in the previous 2 weeks (AOR = 3⋅47, 95 % CI 1⋅34, 8⋅99), having a family member with malnutrition (AOR = 4⋅52, 95 % CI 1⋅45, 14⋅01)., households having greater than five families (AOR = 5⋅33, 95 % CI 2⋅08, 13⋅66), mothers who cannot read and write (AOR = 3⋅66, 95 % CI 1⋅27, 10⋅56), mothers who have the lowest decision-making autonomy (AOR = 5⋅67, 95 % CI 2⋅26, 14⋅27), mothers who did not wash their hands at all critical time (AOR = 7⋅23, 95 % CI 2⋅74, 19⋅07), households who did not feed child animal source food (AOR = 7⋅13, 95 % CI 1⋅98, 25⋅59) and as an exception the being married of mother of children (AOR = 0⋅05, 95 % CI 0⋅02, 19) were independently associated with acute malnutrition (Table 5).

**Table 5.** Risk factors associated with SAM in Liban District, Guji Zone Oromia Region, Southeastern Ethiopia

Variables		Acute malnutrition		COR (95 % CI)	AOR (95 % CI
		Control, *N* (%)	Case, *N* (%)		
Family member with malnutrition	Yes	147 (83⋅1)	52 (58⋅5)	3⋅61 (2⋅03, 6⋅71)	4⋅52 (1⋅45, 14⋅01)*
	No	30 (16⋅9)	37 (41⋅5)	1	1⋅00
Any illness within the last 2 weeks	Yes	44 (24⋅9)	42 (47⋅2)	2⋅77 (1⋅62, 7⋅49)	3⋅47 (1⋅34, 8⋅99)*
	No	133 (75⋅1)	47 (52⋅8)	1	1⋅00
WFA category	Underweight	78 (44⋅1)	72 (80⋅9)	4⋅55 (2⋅45, 8⋅45)	11⋅8 (3⋅17, 43⋅89)*
	Moderate	31 (17⋅5)	10 (11⋅2)	2⋅24 (1⋅03, 4⋅84)	2⋅38(0⋅51, 11⋅19)
	Normal	68 (38⋅4)	7 (7⋅8)	1⋅00	1⋅00
Feeding child animal source food	No	47 (26⋅6)	40 (44⋅9)	2⋅48 (1⋅33, 4⋅60)	7⋅13 (1⋅98, 25⋅59)*
	Yes, less frequently	59 (33⋅3)	25 (28⋅0)	1⋅25 (0⋅65, 2⋅39)	1⋅13 (0⋅38, 3⋅38)
	Yes, daily	71 (40⋅1)	24 (26⋅9)	1	1⋅00
Material to feed child	Bottle	34 (19⋅2)	29 (32⋅5)	2⋅17 (1⋅18, 3⋅98)	7⋅06 (2⋅34, 21⋅28)*
	Cup	37 (20⋅9)	18 (20⋅2)	1⋅26(⋅65, 2⋅44)	2⋅52 (0⋅85, 7⋅44)
	Hand	106 (59⋅9)	42 (47⋅2)	1	1⋅00
Maternal marital status	Married	103 (58⋅2)	71 (79⋅7)	0⋅34 (0⋅18, 0⋅61)	0⋅05 (0⋅02, 19)*
	Unmarried	74 (41⋅8)	18 (20⋅3)	1	1⋅00
Maternal decision autonomy	Low level	74 (41⋅8)	57 (64⋅1)	2⋅61 (1⋅54, 4⋅40)	5⋅67 (2⋅26, 14⋅27)*
	Highest	103 (58⋅0)	32 (35⋅9)	1	1⋅00
Maternal educational status	Illiterate	120 (67⋅0)	73 (82⋅1)	2⋅26 (1⋅21, 4⋅21)	3⋅66 (1⋅27, 10⋅56)*
	Literate	57 (32⋅2)	16 (17⋅9)	1	1⋅00
Handwashing practice	Not at all critical time	52 (29⋅4)	46 (51⋅6)	2⋅62 (1⋅56, 4⋅42)	7⋅23 (2⋅74, 19⋅07)*
	At all critical time	125 (70⋅6)	43 (48⋅4)	1	1⋅00
Family size	>5	36 (20⋅3)	43 (48⋅3)	3⋅92 (2⋅26, 6⋅78)	5⋅33 (2⋅08, 13⋅66)*
	≤5	141 (79)	46 (51⋅6)	1	1⋅00

The number “1” refers to references.

* Variables those were significantly associated with the *P*-value are less than 0⋅05.

## Discussion

The aim of the present study was to identify determinants of acute malnutrition in the pastoral community of Liban District, Guji Zone. Accordingly, we determined that the occurrence of SAM was significantly associated with household family size, maternal educational level, maternal autonomy on decisions, underweight child, child care practices like feeding animal sources, bottle feeding, other family members with malnutrition and poor handwashing practice of mothers.

Children of households that have greater than five family sizes had developing SAM than children of households having less than five family sizes in the present study. This may be attributed to the liability of the children to overcrowding, lower child care practices and child health services. This finding agrees with the study done in western Oromia hospitals and the study done in Bule Hora, Southeastern Oromia, which is a similar setting with the present study area^(5,21)^.

The other socio-demographic factor that was found to be determinant of SAM was maternal education. This may be due to the better quality of child care of relatively educated mothers than mothers who cannot read and write. This finding is similar to the study done in Hadiya zone, Shashigo woreda with a similar design and community setting^(5,22–25)^. Similarly, in this study mother's autonomy in decision-making is significantly associated with SAM among less than the age of 5-year children. Children whose mothers were not autonomous in decision-making were more malnourished than those children whose mothers were autonomous in decision-making. This result is consistent with studies conducted in Shashigo woreda of SNNPR and Dolo Ado woreda of the Somali region^(26,27)^. This could be explained by the fact that the provision of joint care by biological parents requires joint decisions on the cares to their children in order to improve children's nutritional status. Such decision might also require women's autonomy to participate in the decision-making process of the household equally with the men. This result also supports the current policy of government in which empowering women, women education and increasing influence of women have significant impacts on the health of the family and the community.

The present study also showed that mothers who had not practiced handwashing at all critical points associated with acute malnutrition. This finding also agrees with the study findings of the study in western Oromia, Matakal woreda of Northwest Ethiopia. This may be attributed to the potential handwashing at all critical times, especially after toilet, before preparing food, before meal, before breastfeeding and after cleansing child faeces, which prevents a number of infectious diseases and contributes to reduce malnutrition^(28,29)^.

In the present study, being underweight had a chance to develop SAM than those children having normal weight relative to age. In fact, the cause of SAM is multi-factorial that chronic malnutrition could exacerbate due to the current shortage of food intake and acute infections. Thus, the present study signifies that being underweight is more likely to malnourished than normal weight among children under 5 years of age. In a study in Dolo Ado woreda of the Somali region, it was found that the risk factors for being underweight and being wasted are identical^(30)^.

In another hand, the present study described morbidity status of the child with diarrhoea and fever 2 weeks preceding the study is significantly associated with acute malnutrition of the children. Cases had more history of diarrhoea than the controls. This can be due to excessive loss of fluids and electrolytes, loss of appetite and lack of absorption of food in the intestine due to high motility of the intestine during diarrhoea episodes. A similar finding was seen in the studies done in Dolo Ado, western Oromia and Hadiya zone^(5,30–32)^. In addition, children who were previously malnourished had developed acute malnutrition than those who have no previous malnutrition. This may be due to the fact that in households where there is food insecurity and poor child care practiced, there is a likely occurrence of repeated malnutrition^(5,30–36)^.

### Limitations

The study relied on respondents’ self-reported information, which was prone to recall bias and social desirability bias. It is difficult to establish the correct temporal relationship between exposure and outcome of interest which may be attributed to the design itself.

## Conclusions

The present study identifies different determinants of SAM in the Liban District. The determinants found were family size, maternal illiteracy, underweight, not feeding child animal source, bottle feeding, illness within the last 2 weeks and poor handwashing practice of mothers which were independent predictors of acute malnutrition.

To improve child nutrition, the promotion of better child-caring practices by improving the practice of parents on appropriate infant and young child feeding practices, particularly the optimal complementary feeding practices involving a variety of food, should be emphasised. Strengthen intervention like the prevention of diarrhoeal disease may include activities such as the community-targeted promotion of hygiene, handwashing at critical times where mothers/caregivers need to wash their hands before preparing food, before feeding child or breastfeeding and after visiting of toilet or disposing of child faeces.
